# Modification of betanodavirus virulence by substitutions in the 3' terminal region of RNA2

**DOI:** 10.1099/jgv.0.001112

**Published:** 2018-07-24

**Authors:** Sandra Souto, José G. Olveira, Carlos P. Dopazo, Juan J. Borrego, Isabel Bandín

**Affiliations:** ^1^​ Departamento de Microbiología y Parasitología, Instituto de Acuicultura, Universidade de Santiago de Compostela, 15706 Santiago de Compostela, Spain; ^2^​ Departamento de Microbiología, Facultad de Ciencias, Universidad de Málaga, 29071 Málaga, Spain

**Keywords:** reassortant betanodavirus, RNA1, RNA2, interaction, virulence attenuation

## Abstract

Betanodaviruses have bi-segmented positive-sense RNA genomes, consisting of RNAs 1 and 2. For some members of the related genus alphanodavirus, the 3′ terminal 50 nucleotides (nt) of RNA2, including a predicted stem-loop structure (3′SL), are essential for replication. We investigate the possible existence and role of a similar structure in a reassortant betanodavirus strain (RGNNV/SJNNV). In this study, we developed three recombinant strains containing nucleotide changes at positions 1408 and 1412. Predictive models showed stem-loop structures involving nt 1398–1421 of the natural reassortant whereas this structure is modified in the recombinant viruses harbouring point mutations r1408 and r1408–1412, but not in r1412. Results obtained from infectivity assays showed differences between the reference strains and the mutants in both RNA1 and RNA2 synthesis. Moreover, an imbalance between the synthesis of both segments was demonstrated, mainly with the double mutant. All these results suggest an interaction between RNA1 and the 3′ non-coding regions (3′NCR) of RNA2. In addition, the significant attenuation of the virulence for Senegalese sole and the delayed replication of r1408–1412 in brain tissues may point to an interaction of RNA2 with host cellular proteins.

## Introduction

Betanodaviruses, the causative agents of a highly infectious fish disease known as viral nervous necrosis (VNN), are members of the family *Nodaviridae*, which also includes alphanodaviruses that naturally infect insects [1].

Betanodaviruses are small, non-enveloped icosahedral viruses with bipartite positive-sense genomes. The larger genome segment, RNA1 (3.1 Kb), encodes the viral RNA-dependent RNA polymerase (RdRp), also known as protein A, while the smaller genome segment, RNA2 (1.4 Kb), encodes the only structural protein. RdRp replicates both genomic RNAs and also transcribes RNA3, a non-encapsidated subgenomic RNA that is co-terminal with the 3′ end of RNA1 and encodes B2 protein, an inhibitor of cell RNA silencing, and B1, an anti-necrotic death factor [2, 3]

Betanodavirus isolates are currently classified into four different genotypes: striped jack nervous necrosis virus (SJNNV)-type, tiger puffer nervous necrosis virus (TPNNV)-type, barfin flounder nervous necrosis virus (BFNNV)-type and redspotted grouper nervous necrosis virus (RGNNV)-type, on the basis of phylogenetic analysis of the RNA2 variable region [4]. However, in Southern Europe in recent years the existence of a natural reassortment between RGNNV and SJNNV genotypes (in both RNA1/RNA2 forms: SJNNV/RGNNV and RGNNV/SJNNV) has been observed in viral isolates obtained from Senegalese sole (*Solea senegalensis*), gilthead sea bream (*Sparus aurata*) and sea bass (*Dicentrarchus labrax*) [5–7]. Reassortant strains isolated from diseased farmed Senegalese sole and gilthead sea bream in the Iberian Peninsula showed an RNA1 typed as RGNNV and a SJNNV-type RNA2, and exhibited differences at both the genomic and protein levels as well as in the 3′ non-coding region (NCR) when compared to the reference strains of each genotype [5, 8]. To date, only these reassortant strains have been associated with natural infections in Senegalese sole and sea bream [5, 9], which suggests that the genomic changes caused by reassortment might favour the colonization of these fish species.

Genome replication is a critical step in virus life cycles. Positive-strand RNA viruses replicate their genomes by viral replicase complexes consisting of virus-encoded RNA-dependent RNA polymerase and other proteins (viral or host derived) [10]. The replication process requires not only the recognition of specific sequences and structural features by viral polymerases, but also the existence of signals in the genomic RNAs, such as cis-acting elements, that are essential for replication. These elements may be located in protein-coding regions and/or in the 5′ and 3′ NCRs of the viral genome [11–18]. In alphanodaviruses, it has been reported that RNA replication is dependent on cis-acting elements at the 5′ and 3′ termini, but it is also governed by internally located cis-acting elements on both genomic RNAs [19–23]. Recent reports have suggested that stem-loop structures located at the 3′ end on RNA2 (3′SL) form part of an essential cis-acting signal for the replication of this segment [24]. Stem-loop (SL) structures have also been predicted in this region in two betanodaviruses, SJNNV and G*reasy grouper nervous necrosis virus* (GGNNV) [25].

In the present report, we performed RNA2 structure predictions to investigate the possible existence of 3′SL structures in reassortant betanodavirus strains (RGNNV/SJNNV) isolated from sole, which show differences with the SJNNV-type reference strain SJNag93 at nucleotides 1408 and 1412 [8]. Therefore, we examined the predicted effect of these two mutations on molecule conformation. In addition, the effect of these two mutations on infectivity *in vitro*, as well as on virulence for fish, has been analysed.

## Results

### Predicted 3′SL structure in betanodavirus reassortant strains

**Fig. 1.**
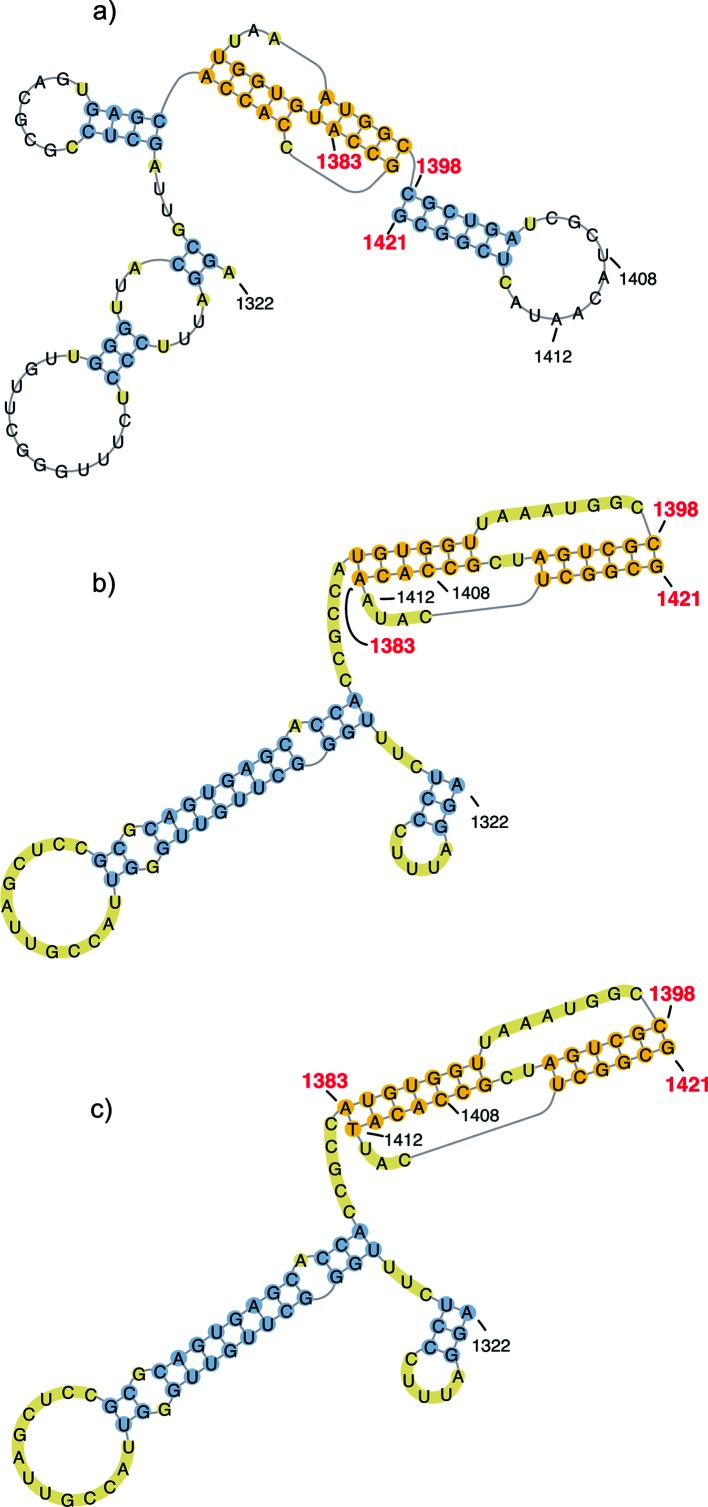
RNA structure. Predictions of the 3′ terminal 100 nt of RNA2 for wt160 and recombinant strains harbouring point mutations were generated by HotKnots 2.0 [46] and mfold [47], and the figure was produced using PseudoViewer software. (a) A stem-loop structure (3_SL) predicted to form at the 3′end of wt160 RNA2 comprising nt 1398–1421. (b and c) Pseudo-knot predicted for r1408 (b) and for r1408–1412 (c) comprising nt 1383–1421.

For the natural reassortant betanodavirus strain wt160 (wild-type strain), the software predicted a pseudo-knot in the 3′ termini followed by a stem-loop structure involving nt 1398–1421 (Fig. 1a). This structure was modified in the predictions obtained for the recombinant viruses harbouring point mutations: r1408 and r1408–1412. In these recombinant strains a pseudo-knot structure involving nt1383–1421 was identified (Fig. 1b, c). However, for the mutant r1412 the predictive model was identical to that of the wt strain.

### Infectivity *in vitro*

To investigate whether the point mutations in the 3′NCR of the RNA2 segment influence the efficiency of viral replication *in vitro*, RNA synthesis and viral production were studied in E-11 cells.

#### RNA synthesis

**Fig. 2.**
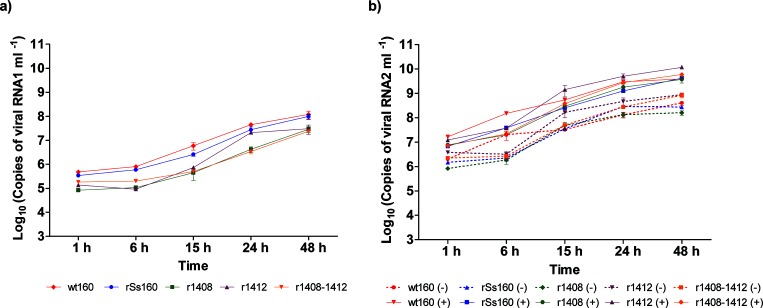
RNA synthesis in cell lysates. Quantitative real-time PCR (qPCR) was used to determine the number of genome copies per millilitre of cell lysates at different time points. Monolayers of E-11 cells in 48-well plates were infected with different viruses at a m.o.i. of 0.01. The cells were scraped and harvested at 6, 15, 24 and 48 h post infection (h p.i.). (a) Quantification of RNA1 copy numbers; (b) quantitation of ±strand RNA2. Values are expressed as means±sd (*n*=3).

As shown in Fig. 2, both genomic segments were detected as early as 1 h p.i. of the cell monolayers. Quantification of RNA1 from cell lysates showed initial values ranging from 8.3×10^4^ to 4.8×10^5^ copy number ml^−1^ and from 1 to 6 h p.i., a slight increase was observed in the reference strains (Fig. 2a). The RNA1 yield was substantially increased from 6 to 48 h p.i. in the reference strains, reaching 9.8×10^7^–1.2×10^8^ copy number ml^−1^(Fig. 2a), whereas in the recombinant strains harbouring point mutations, especially r1408 and r1408–1412, the RNA increase was lower. Differences of more than 1 log were observed when compared to the wt160 strain from 15 to 24 hp.i. At 48 h p.i. these differences were reduced to 0.6–0.7 logs. To determine the duration of the exponential phase of RNA1 synthesis, its kinetics was analysed by regression analysis (results are shown in Table S1, available in the online version of this article). The exponential phase appeared to start very early (1 h p.i.) in both reference strains, whereas in both single-point mutants the beginning of this phase was delayed until 6 h p.i., and in the double mutant no real exponential – lineal – phase was confirmed.

Since the point mutations were targeted at RNA2, we expected to observe more alterations of its kinetics of synthesis; therefore, we decided to assess the RNA production of both the positive strand (genomic RNA) and negative strand generated as an intermediate during the RNA2 replication process. Sense and anti-sense RNA transcripts were used to assess the strand specificity of the method. Neither false priming in the RT nor cross-amplification was detected. Amplification of the wrong strand was checked by carrying out the negative/positive-strand RNA quantitation protocol in the presence of both strand-synthetic RNAs. As with RNA1, RNA2 was detected as early as 1 h p.i. (Fig. 2b), reaching its highest values (3.7×10^9^–1.2×10^10^ copy number ml^−1^ at 48 h (Fig. 2b); and, as before, the exponential phase of RNA2(+) synthesis was between 1 and 24 h p.i., but in this case present in all strains. However, for RNA2(−) production from 1 to 6 h. only exponential kinetics behaviour with a significant increase (*P*<0.0001) was observed in the wt strain, whereas in the recombinant strains the beginning of the exponential phase was delayed by up to 6 h (Table S1). The ratio of positive- to negative-sense RNA ranged from 5 : 1 at 1 h p.i. to 15 : 1 at 48 h p.i. in wt160, and from 3 : 1 to 23 : 1 in the mutant strains (Fig. 2b).

No significant differences (below 1 log of titre) were observed among the different strains. Comparison of the production of both genomic segments showed that the number of RNA2 copies was higher than those of RNA1 at all time points analysed. It is worth noting that the differences observed were higher in the mutant strains, i.e. at the different time points the ratio of RNA2 to RNA1 ranged from 20 : 1 to 189 : 1 in wt160, whereas in r1408–1412 it ranged from 40 : 1 to 815 : 1.

**Fig. 3.**
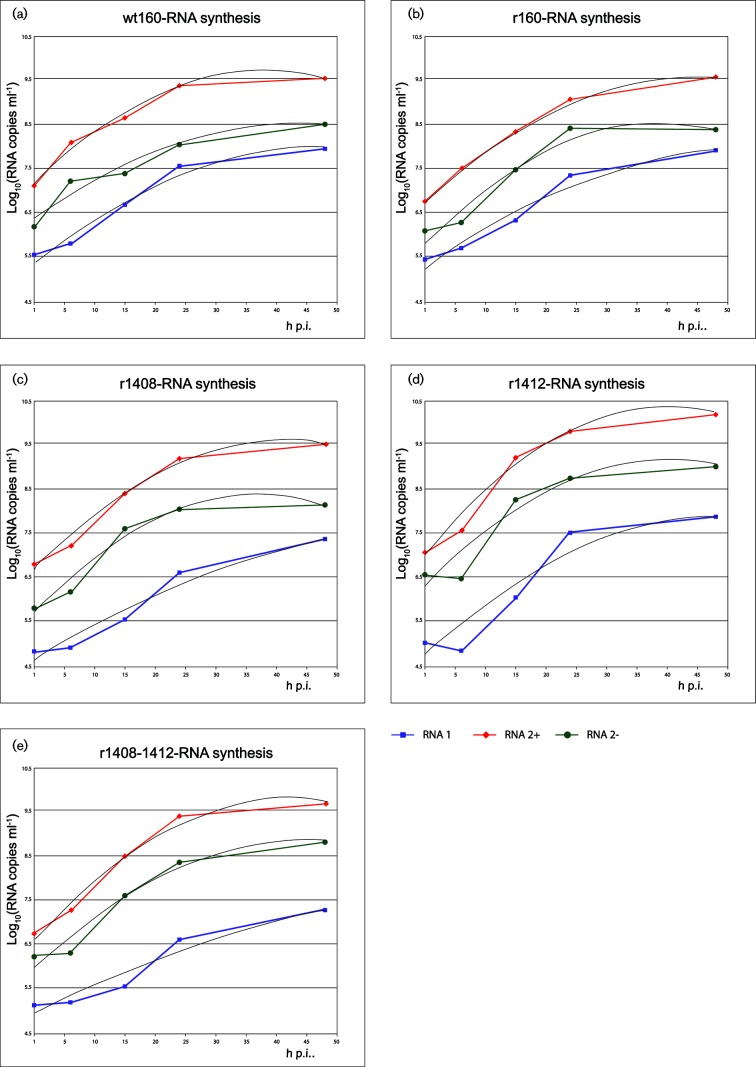
RNA synthesis in cell lysate regression curves. Regression analysis (second-degree polynomial) of the curves described in Fig. 2 was applied to both RNA1 and RNA2 (plus and minus strands) synthesis data (3 replicas per time point). The regression curves are represented in this figure, and their corresponding equation and R^2^ are shown in Table S2.

However, we needed to be more precise since, on the one hand, we were aiming to determine whether there were significant differences among reference strains and mutants, and on the other to demonstrate that there was a balance between the reference strains kinetics synthesis of RNA1 and RNA2, and of RNA2(+) and RNA2(−), and whether this balance was destroyed in any of the mutants (Fig. 3). Therefore, all RNA synthesis curves were adjusted to second-degree polynomial functions and, as shown in Table S2, except for some specific cases, their reliability was significant (R^2^>0.95).

Comparison between RNA2(+) and RNA2(−) curves revealed that in most cases no correlation could be determined (*P*>0.05); only in the case of wt160 were differences between both slightly significant (*P*=0.0475) (Table S2). When the analysis was extended to RNA1/RNA2, a correlation between both segments was observed in the reference strains (wt160 and r160), and a significant lack of correlation was demonstrated in two of the mutants: r1408 and r1408–1412.

No differences in RNA1 synthesis were observed among the strains (Table S3). However, significant differences in RNA2(+) existed between the reference strains and mutants r1412 and r1408–1412.

#### Viral replication kinetics

**Fig. 4.**
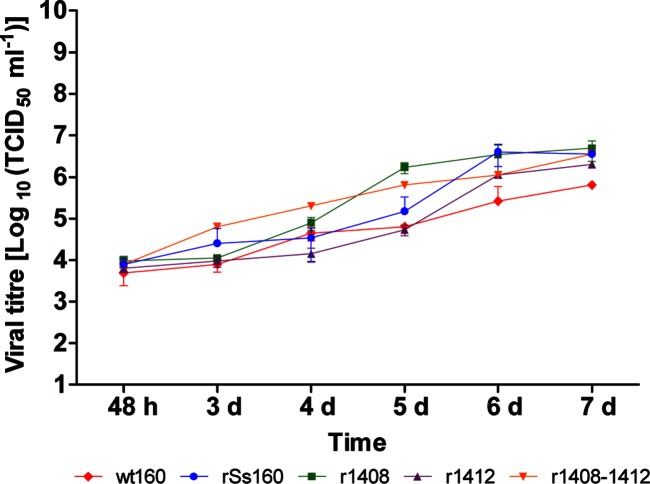
Viral production in cell supernatants. Monolayers of E-11 cells in 25 cm^2^ flasks were infected with various viruses at a m.o.i. of 0.01 and supernatants harvested at the indicated time points. Infectious titres were determined by the end-point titration method and are expressed as mean TCID_50_ ±sd (*n*=3) per millilitre of cell supernatant.

Culture supernatants harvested at 6, 15 and 24 h p.i. yielded no cytopathic effect when inoculated into E-11 cells; therefore, to assess viral production and construct the growth curve, the study was initiated at 48 h p.i. As observed in Fig. 4, at 2 days p.i. all strains showed similar titres although the wt strain showed lower viral production than the recombinant strains. Among the mutant strains some differences were observed from the third day. These differences were more obvious at days 4 and 5 p.i., when the r1412 strain appeared to replicate more slowly than the other mutants (showing a titre 1.0–1.5 log below), but they were reduced at day 6. Finally, at 7 days p.i. all recombinant strains showed a similar titre. Unfortunately, since most of the curves (except mutant r1408–1412) were not significantly (R^2^<0.95) adjusted, either to a linear or a second-degree polynomial function (Fig. S1), they could not be statistically compared to confirm those differences or similarities.

**Fig. 5.**
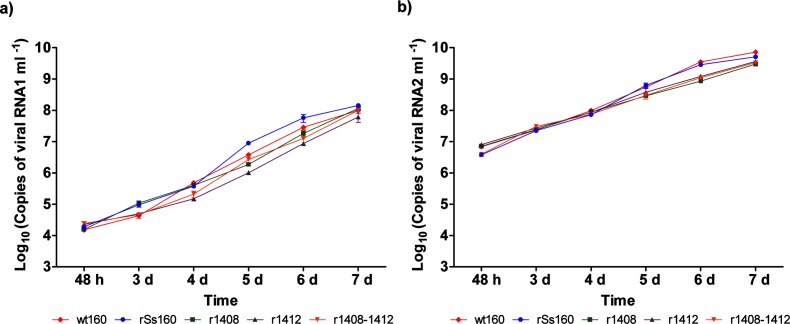
Viral RNA production. Supernatants harvested at the indicated time points in the viral replication experiment were used to quantify the number of genome copies per millilitre of supernatant by RT-qPCR. (a) RNA1 copy number; (b) RNA2 copy number. Values are expressed as means±sd (*n*=3).

Viral production was also investigated in terms of extracellular viral RNA (Figs 5, S2 and S3). At 48 h p.i. extracellular RNA 1 quantification yielded 1.9×10^4^ copies ml^−1^ (average value) and no differences were observed among the strains. However, on subsequent days as extracellular RNA1 increased, slight differences (<0.5 log) were detected between the reference and mutant strains (Fig. 5a). Interestingly, at 5 days p.i. the greatest difference was observed between the mutant r1412 and the r160 strain. However, at 7 days p.i., when CPE was extensive, RNA1 yield was similar in all strains (average value 1.0×10^8^ copies ml^−1^). Comparison of RNA1 production kinetics revealed no significant differences among the strains (Fig. S2).

Extracellular RNA2 reached higher values than RNA1, copy numbers ranging from 5.9×10^6^  to 4.4×10^9^ copies ml^−1^ (average values at days 2 and 7 p.i., respectively). Detailed analysis indicated that the double mutant and r160 showed slightly lower production than the other strains at 48 h p.i. Subsequently, all strains displayed very similar values and a difference of around 0.5 log was observed between the reference strains and r1408 only at 6 days p.i. However, when the RNA2 production curves were analysed, significant differences were observed between those corresponding to the reference strains and r1408 and r1412.

### *In vivo* infection

#### Virulence for Senegalese sole

**Fig. 6.**
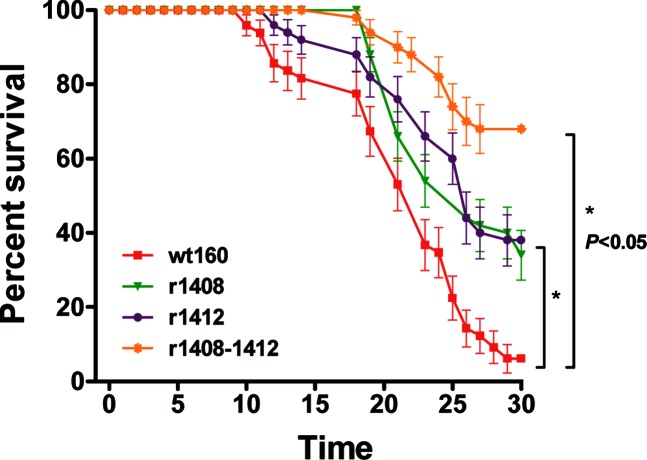
Virulence of viral strains for Senegalese sole. The curves represent fish survival rates after infection by immersion with wt160 and mutants r1408, r1412 and r1408–1412. Values are expressed as means±sd (*n*=3). Asterisks denote significant differences (*P*<0.05).

In the experimental challenges performed by immersion, 95 % mortality was observed at 30 days p.i. in the groups infected with wt160. Survival percentage increased in sole infected with different recombinants harbouring point mutations, especially in those groups infected with the double mutant. In fish infected with recombinants harbouring one single mutation (r1408 and r1412) survival was around 40 %, whereas in the groups infected with the double mutant r1408–1412 the survival rate increased up to 68 % (Fig. 6). Mortality was recorded earlier in wt160-infected fish (at 10 days p.i.), followed by individuals infected with r1412 (at 12 days p.i.). Mortality in groups infected with r1408 and the double mutant r1408–1412 were first recorded at 19 and 18 days p.i., respectively. Signs of disease (loss of appetite, hyperactivity and erratic swimming) were observed in all groups, although these were always more severe in groups infected with the wt strain. The sequencing of viruses re-isolated in E-11 cells after infection became established showed the existence of the corresponding mutations.

#### RNA replication in sole brain

**Fig. 7.**
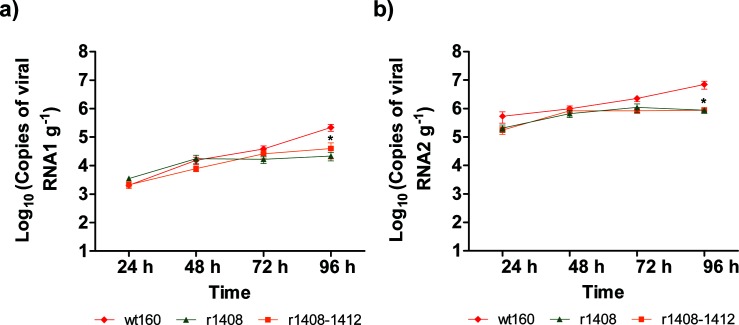
RNA replication in sole brain. Sole challenged by immersion with wt160 and the mutants r1408 and r1408–1412 were euthanized at the indicated time points. The number of genome copies in brain tissue was determined by quantitative RT-qPCR. (a) RNA1 copy number; (b) RNA2 copy number. Each point represents the mean RNA copy number±sd from six fish.* *P*<0.05 compared to value for wt160.

All strains were detected in brain tissue at 24 h p.i. with a similar RNA1 copy number (Fig. 7a). The RNA1 values in the wt strain increased steadily from 2.0×10^3^ to 2.2×10^5^ copies g^−1^ at 96 h p.i., reaching a higher viral load than mutants at 96 h p.i. (*P*<0.05), whereas RNA1 yield in the mutants showed a moderate increase at the beginning but reached a plateau after 48 h for r1408, and after 72 h for r1408–1412.

RNA2 quantification revealed a similar picture, the copy number in the wt strain increasing steadily from 5.4×10^5^ copies g^−1^ at 24 h p.i. to 7.0×10^6^ copies g^−1^ at 96 h p.i. (Fig. 7b). However, in the mutants, after 48 h no significant increase was observed, and at 96 h p.i. there was a significant delay in growth (*P*<0.05).

## Discussion

It has been demonstrated that the 3′ non-coding region of positive-strand viral RNAs is involved in RNA synthesis with different potential roles, namely, in minus strand synthesis (promotion or regulation of access to minus strand origin), in the modulation of translation or in RNA targeting to specific subcellular sites [26–29].

In the present study, a stem-loop structure was predicted at the 3′ termini of RNA2 of reassortant betanodavirus strain wt160 comprising nt 1398–1421, which is in agreement with previous predictive approaches for the SJNNV strain [25]. In alphanodaviruses it has been reported that the 3′ terminal 50 nucleotides of RNA2 contain a *cis*-acting replication signal capable of directing the replication of this segment. In experiments performed with Flock house virus (FHV) and Nodamura virus (NoV), replacement or deletion of this *cis*-acting signal has demonstrated that it is RNA3-dependent and includes a 3′SL structure, respectively [19, 24]. In addition, deletion of the 3′SL structure in NoV markedly affected RNA2 replication [24]. Although we did not perform a complete deletion of the 24 nt contained in the putative 3′SL structure, predictive modelling indicated either disruption or deformation of this structure due to the substitution of nt 1408 or nt 1408 and 1412. We expected these changes to have a dramatic effect on the synthesis of either the positive or negative RNA2 strands. However, although it is true that significant differences between reference strains and mutants were observed in RNA2(+) synthesis, we must recognize that these were not as substantial as expected. Moreover, results with the mutant r1412 call into question whether or not the proposed 3′SL is the mechanism by which these nucleotides affect viral function. It could be because the mutations involved too few nucleotides (despite the predicted structure disruptions) or because these predictions were not sufficiently accurate, since predictive software estimates the structures based on conditions that may not be appropriate for RNAs in living cells. Alternatively, it could be because the cis-acting signal required for RNA2 replication is not located in the stem-loop structure in these betanodavirus strains. Further studies including nuclease mapping will be necessary to elucidate this point.

With regard to RNA1, although no significant differences in its kinetics of synthesis were observed among the strains, differences of more than 1 log between the reference strains and both recombinants harbouring point mutations (r1408 and the double mutant r1408–1412) were noted at 24 h p.i. Furthermore, in these strains the ratio RNA2:RNA1 was up to sixfold higher than in wt160 or r160, and an imbalance between the synthesis of both segments was demonstrated, mainly with the double mutant (*P*<0.0001). Interestingly, this was the strain yielding the lowest mortality *in vivo*, which may indicate that disruption of the 3′SL structure affects its survival in fish by affecting its replication. However, the fact that both single mutants also showed reduced mortality and that r1412 showed no apparent alteration in 3′SL suggests that minor changes in 3′NCR have important effects on virulence *in vivo*.

Nodaviruses, like other segmented viruses, are required to coordinate or modulate the replication of the two genomic RNA segments. In alphanodaviruses it has long been known that the synthesis of RNA3, encoded by RNA1, is suppressed by the replication of RNA2 [30, 31], and more recently it has been suggested that RNA3 could act as a transactivactor of RNA2 replication [19, 32, 33]. In addition, research has also revealed that base-pairing within RNA1 could support RNA2 replication in *trans* [23]. Our results suggest the existence of such an interaction between RNA1 and the predicted 3′SL structure in the NCR of RNA2, but unlike that reported by [23], this would affect RNA1 synthesis, in addition to the balance between RNA1 and RNA2 synthesis. Further studies should be carried out to determine whether an intermolecular interaction or a long-distance *cis* or *trans* interaction is established.

Virulence for sole was clearly affected by the substitutions in the 3′ NCR of wt160 RNA2. Survival barely reached 5 % in fish infected with the wt strain, whereas in the groups challenged with the recombinant strains harbouring single mutations, survival reached 40 %. However, the mutation of both positions led to the highest increase in survival, to almost 70 %. Reduction of virulence in the mutants could have been caused by the observed effects on RNA1–RNA2 interaction. Inadequate RNA1 production can have deleterious consequences on viral progeny. However, viral infectivity in E-11 cells, measured as TCID_50_ titres, showed no significant differences between these mutants and wt160 or r160. These findings suggest that the 3′NCR nucleotide sequence could interact with host cellular proteins required for viral replication, as previously reported for different positive-strand RNA viruses such as Japanese encephalitis virus [34–36], dengue virus [37, 38], hepatitis C virus [39] and Norwalk virus [40]. Analysis of viral replication in sole brain tissue indicated that, although the mutants reached the brain concurrently with the wt strain, and even at slightly higher levels, thereafter, their replication was very slow. This impairment of mutant replication in brain tissue could confirm the role of the interaction of RNA2 with host proteins in virulence attenuation, but it could also involve a differential susceptibility to host immune defences. The fact that no differences were observed between replication of the wt strain and the mutants in E-11 cells may be due to the participation of different cellular proteins in the RNA replication process. We previously postulated that receptors present in this cell line may be different to those which mediate the entry of betanodavirus in fish neuronal cells [8], as demonstrated in rabies virus [41].

Experimental studies using primary neuron cultures from sole are in progress to further analyse this interaction and the host proteins involved.

In summary, the data obtained in this study appear to support the key role in betanodavirus replication of 3′NCR, and for the specific nucleotides 1408 and 1412, especially *in vivo*. However, the secondary structure predictions are contradictory, because although the 3′SL structure was modified in mutants r1408 and r1408–1412, no changes were observed in r1412. There are two possible explanations for these results: either 3′SL is not the mechanism responsible for the changes observed in viral replication and virulence, or the predictive models were not sufficiently accurate. Further studies will be necessary to clarify this issue.

## Methods

### Cell cultures, viruses and titration

In the present study, two betanodavirus strains were used as reference: the natural reassortant with an RGNNV RNA1 and an SJNNV RNA2 SpSsIAusc16003 (abbreviated to wt160) [5] and the recombinant virus r160, a virus produced by reverse genetics with a genome sequence identical to wt160 [8]. In addition, three recombinant strains harbouring point mutations as described below were also employed. All strains were grown in E-11 cells, clone-derived from the SSN-1 cell line which is a mixed-cell population obtained from striped snakehead (*Channa striatus*) and persistently infected with a C-type retrovirus (SnRV) [42, 43]. Cells were maintained in L-15 Leibovitz (Lonza) medium supplemented with penicillin (100 units ml^−1^ streptomycin (100 mg ml^−1^) and 2 % fetal bovine serum (FBS, Lonza), at 25 °C. After each experiment the identity of viral recombinants was conﬁrmed by 3′ RACE (System for Rapid Amplification of cDNA Ends) as described in [8] and sequencing (GATC Biotech).

For viral titration, the endpoint dilution method was performed in 96-well plates (Sarstedt), and the titres expressed as the viral dilution infecting 50 % of the cell cultures (TCID_50_) following the methodology described in [44].

BSRT7/5 cells [45], kindly provided by Dr K. K. Conzelmann (Ludwig-Maximilians-Universität Munich), were maintained in Dulbecco’s modified Eagle’s medium (DMEM; Lonza) supplemented with 10 % fetal bovine serum (FBS), glutamine (2 mM l^−1^, Lonza), penicillin (100 U ml^−1^) and streptomycin (100 mg ml^−1^) at 37 °C in a 5 % CO_2_ humidified chamber. Geneticin (G418, 1 mg ml^−1^ final concentration) was added every two subcultures.

### Recovery of recombinant viruses

The strains r1408, r1412 and r1408–1412 – recombinants with RNA1 identical to wt160 and a wt160 RNA2 with point mutations – were generated by reverse genetics as described previously [8]. Briefly, the plasmid pBS160R2 [8], which contains the full-length cDNA of the RNA2 genome sequence from the wt160 strain, was subjected to site-directed mutagenesis using a QuickChange Multi Site-Directed Mutagenesis kit (Agilent) according to the manufacturer’s instructions, and the specific primers (lower case letters in bold indicate the desired nucleotide change): Mut1408- 5′GCTGATCGC**c**ACAATACTCGGCGGGGTC GGCATGGC3′, Mut1412- 5′GCTGATCGCTACA**t**TACTC GGCGGGGTCGGCATGGC3′ and Mut1408-1412- 5′GC TGATCGC**c**ACA**t**TACTCGGCGGGGTCGGCATGGC3′.

The resultant plasmids contained nucleotide changes (T1408C and A1412T) in the 3′ NCR of RNA2 (SpSsIAusc16003, GenBank accession number NC_024493.1). BSRT7/5 were transfected, as previously described in detail [8], with 1 µg of each of the two plasmids encoding full-length cDNA copies of the viral wt160 RNA1 (pBSRNA1) [8] and RNA2 (1 µg of either pBS160R2_1408, pBS160R2_1412 or pBS160R2_1408–1412), and 6 µl Lipofectamine2000 (Invitrogen). After 7 days, cells were suspended in the supernatant medium by scratching the wells and were then subjected to freezing/thawing and clarification by centrifugation. The supernatants were subjected to several passages for 7 days in E-11 cells until CPE was observed. The presence of the desired mutations was confirmed by sequencing (GATC Biotech).

### RNA structure predictions

RNA structure predictions were performed on the terminal 100 nucleotides of the 3′ end of RNA2 of each strain, using HotKnots 2.0 [46] and Mfold [47] software at 37 °C as the default temperature. These programmes were chosen because they were most similar to those used previously for alpha and betanodaviruses prediction [24, 25].

### Infectivity *in vitro*

To study the effect of the mutations on viral replication, two different assays were carried out in E-11 cells comparing the replication kinetics of all recombinant viruses to the wild-type reassortant wt160. At the end of each experiment the maintenance of mutations in the recombinant strains was confirmed by sequencing.

#### RNA synthesis kinetics within cells

The genome synthesis at early infection (1–48 h p.i.) was determined by inoculating cells in 48-well plates (Sarstedt) with each viral strain. The cells were infected at a m.o.i. of 0.01 and subsequently washed, overlain with fresh medium and incubated at 25 °C. At 6, 15, 24 and 48 h p.i. supernatants from three wells per strain were removed and the cells were scratched, suspended in 0.5 ml of L-15 medium and harvested separately. Quantitative real-time PCR (qPCR) was used to determine the number of genome copies from cell lysates at the different time points, as further indicated. Extension of the exponential phase was established using regression lines and/or significant increase in RNA levels. First, we assessed whether the growth of each strain adjusted to a line, using regression analysis from the first time point to the last; if the resultant regression line was not significant (R^2^>0.95), the regression analysis was performed from the second time point. When no significant regression line was found, Bonferroni’s multiple comparison was performed between consecutive time points to determine the existence of a significant increase in RNA load.

#### Viral and extracellular RNA production in cell supernatants

To study viral production, 25 cm^2^ (Sarstedt) flasks were infected with the various vir at m.o.i. of 0.01. After 1 h adsorption, the inoculum was removed and the monolayers were washed three times and covered with L-15. The infected cells were incubated at 22 °C and monitored daily for cytopathic signs. Samples from culture supernatants were harvested at 6, 15, 24 and 48 h p.i., and then on a daily basis until the cytopathic effect was extensive. All samples were stored at −80 °C until use. Infectious virus and viral RNA were quantified by end-point titration in E-11 cells and RT-qPCR, respectively.

### Infectivity *in vivo*: experimental challenges

Experimental infections in Senegalese sole were performed to assess the virulence of the various recombinant strains. Juvenile sole (mean weight 2 g) were obtained from a commercial fish farm and maintained at the fish facilities of the Universidade de Santiago de Compostela. The fish were fed dry commercial pellets *ad libitum* daily. All efforts were made to minimize animal suffering. The fish were placed in opaque tanks containing sea water and acclimated at the experimental temperature (22 °C) for 10 days. Before experimental infection, 10 fish were sacrificed with an anesthetic overdose (MS-222, Sigma-Aldrich) and used for both the diagnosis of bacterial pathogens and four regular viral agents: infectious pancreatic necrosis virus (IPNV), infectious haematopoietic necrosis virus (IHNV), viral haemorrhagic septicaemia virus (VHSV) and betanodavirus, as described in [48].

#### Virulence assays

Groups of 30 juvenile sole were infected with each of the three mutants and also with the wild strain (wt160), in triplicate. The virulence of r160 has previously been shown to be identical to that of wt160 [8], and therefore this strain was not included in this assay to minimize the number of fish to be infected. All challenges were performed by bath immersion at a virus concentration of 10^5^ TCID_50_/ml for 3 h, with strong aeration. Control fish (*n*=10) were handled as for the infected groups and L-15 medium was used for mock infection. Mortality and clinical signs were recorded daily, and dead fish were removed. Both sampled and surviving fish were euthanized using an overdose of MS-222. Brain tissue (in pools of five) were analysed by cell culture and RNA quantification.

#### RNA synthesis in brain tissue

Three further groups of 24 sole were placed in three separate tanks and infected with mutants r1408 and r1408–1412, and with the wild strain (wt160), under the conditions described above. A control group of 12 fish was also included. Six fish from each tank were sampled at 24, 48, 72 and 96 h p.i. and brain tissues were analysed individually by RT-qPCR to quantify the level of viral RNA synthesis as described below.

### Processing of samples

Brain tissues were homogenized in Earle's balanced salt solution (1 : 10 for individual brains and 1 : 5 for pooled samples) supplemented with antibiotics (1000 IU ml^−1^ penicillin, 1000 µg ml^−1^ streptomycin, 500 µg ml^−1^ gentamycin and 500 µg ml^−1^ partricin). The samples were centrifuged at 3000 ***g*** for 15 min at 4 °C. When tissue pools where used, the resulting supernatants were split into two aliquots: one stored at −80 °C for later use in RT-qPCR and the other used at that time for cell culture.

### Virus isolation

Cell lysates from the *in vitro* experiments and tissue homogenates were incubated for 24 h at 4 °C, clarified at low-speed centrifugation and subsequently the supernatants inoculated onto monolayers of E-11 cells in 24-well plates at final dilutions of 1 : 100 and 1 : 1000. The plates were incubated at 25 °C and monitored for cytopathic effect (CPE) for 7 days.

### RNA1 and RNA2 quantification

Total RNA was extracted from the samples obtained in the *in vitro* experiments and from tissue homogenates using the EZNA Total RNA I kit (Omega Biotek) in accordance with the supplier’s protocol. Extracted RNA was reverse transcribed with Superscript IV reverse transcriptase (Invitrogen) using random primers. For qPCR, reactions were processed with 2 µl of cDNA samples in a final volume of 20 µl, using iQTMSYBRGreenSupermix (Bio-Rad) and 200 nM of primers SnodR1 F/R [48] for RNA1 and primers SJCoatR2 F/R: GGATTTCGTTCCATTCTCTTGGG/AATCAATGGGCAACGGTTTGTC for RNA2. Quantification of genome copies was accomplished using standard curves generated from tenfold serial dilutions of recombinant plasmids DNA in the range of 10^1–^10^7^ copies µl^−1^. Reactions were carried out using a CFX96TM Real-Time PCR Detection System (Bio-Rad). Following an initial denaturation/activation step at 95 °C for 15 min, the mixture was subjected to 40 cycles of amplification (denaturation at 95 °C for 15 s, annealing and extension at 60 °C for 15 s). Data were expressed as mean±sd of copies per millilitre of cell lysate or millilitre of supernatant or grams of brain tissue for RNA synthesis, viral production and *in vivo* experiments, respectively.

### Strand-specific quantification

The tagged primers TAGnodaR2Rv (5′ CAGGCGTTGTCCGTGTTCTAATCAATGGGCAACGGTTTGTC 3′) and TAGnodaR2Fw (5′ CAGGCGTTGTCCGTGTTCTGGATTTCGTTCCATTCTCTTGGG 3′) were used for positive- and negative-sense RNA2 cDNA synthesis, respectively. Before qPCR, the reactions were incubated with 2 U of exonuclease at 37 °C for 30 min followed by 70 °C for 15 min as previously described [49]. Real-time PCR was performed using a Kapa Probe Fast Universal qPCR Kit (Kapa Biosystems) with 100 nM of the specific primer sets SJCoatR2 F or SJCoatR2 R (for positive- or negative-sense stands, respectively) and the TAG primer (CAGGCGTTGTCCGTGTTCT), along with 100 nM of the probe ACCCAACTCGACCTCGCTCCTGCA, which used the fluorescent dye FAM with black hole quenchers (BHQ-1), and 2 µl of cDNA in a final volume of 20 µl. The thermal profile consisted of incubation at 3 min at 95 °C followed by 40 cycles of 15 s denaturation at 95 °C and 30 s annealing and extension at 60 °C. For absolute quantification and evaluation of the sensitivity of the strand-specific qRT-PCR, positive- and negative-sense RNA transcripts were synthetized *in vitro*. A 427 pb-long region from RNA2 of the w160 strain was recovered by RT-PCR by using F2R3 primers [50] and inserted into pGEM-T Easy Vector (Promega). Synthetic positive- and negative-sense transcripts were created by separate *in vitro* transcription reactions using the MAXIscript SP6/T7 Transcription Kit (Invitrogen) according to the manufacturer’s instructions. To synthesize the positive-sense template, pR2f2r3 was linearized with *PstI* enzyme. The RNA transcripts were synthesized using T7 promoter. To synthesize the negative-sense template, pR2f2r3 was linearized with *SacII* enzyme and the *in vitro* transcripts were produced using the promoter SP6. For each reaction, 5 µl of linearized DNA template was incubated at 37 °C for 1 h with RNA polymerase. The transcribed RNA was digested with Turbo DNAase (Ambion) according to the manufacturer’s protocol. The RNA transcripts were further purified by extraction with a High Pure RNA isolation kit (Roche) with an on-column DNase-treatment step following the manufacture’s protocol. The quality and quantity of the RNA transcripts were measured using a NanoDrop ND-100 spectrophotometer (NanoDrop Technology).

The efficiency of qRT-PCR using T7- and SP6-transcribed RNAs, positive and negative strand, respectively, was evaluated using the slope of the amplified curve to determine the copy number of the RNA2 region starting at 5×10^7^ copies.

### Statistical analysis

Statistical analyses were carried out by GraphPad Prism version 6.00 (GraphPad Software, La Jolla, CA). *P*<0.05 was considered statistically significant. *P-*values were adjusted by the Bonferroni method for multiple comparison. In the case of linear regression, to determine whether two or more curves were similar, the slopes and intersections were compared by an F-test. In the case of kinetics adjusted to second-degree polynomial functions (y = B_2_x^2^+B_1_x+B_0_), the parameters B_1_ and B_2_ were chosen for comparison: B_1_ giving the position of the parabola on the x-axis and B_2_ the amplitude of the parabola, and the (x, y) position of its vertex. When the P-values were low (*P*<0.05) for both parameters, the theory that differences are due to random sampling was rejected and therefore both curves were considered different; if differences were demonstrated only for the intersections, the curves were assumed to be distinct but parallel.

## Supplementary Data

Supplementary File 1
