# Supplementary material for: Modification of betanodavirus virulence by substitutions in the 3' terminal region of RNA2
Source: J Gen Virol. 2018 Jul 24;99(9):1210–20. doi: 10.1099/jgv.0.001112 (PMC6230769; doi:10.1099/jgv.0.001112)
Supplement: Supplementary File 1 [file jgv-99-1210-s001.pdf]

Supplementary Files

Table S1.- Regression analysis: Analysis of the exponential phase of RNA synthesis

Strain	RNA	Regression line between 1 and 24h		Regression line between 6 and 24h	
		Equation	R^2	Equation	R^2
wt160	RNA 1	y = 0,6551x + 4,7446	0,96962	y = 0,0973x + 5,2092	0,99998
	RNA 2+	y = 0,6072x + 6,712	0,95066	y = 0,0722x + 7,6059	0,99306
	RNA 2-	y = 0,5425x + 5,8347	0,96270	y = 0,0452x + 6,86	0,93017#
r160	RNA 1	y = 0,6586x + 4,5458	0,96397	y = 0,0928x + 5,0407	0,98276
	RNA 2+	y = 0,7126x + 6,0632	0,99441	y = 0,0842x + 6,9908	0,99775
	RNA 2-	y = 0,6662x + 5,2871	0,91271#	y = 0,1177x + 5,5804	0,99448
r1408	RNA 1	y = 0,6768x + 3,8173	0,94041#	y = 0,0934x + 4,2877	0,97669
	RNA 2+	y = 0,7321x + 5,9927	0,96600	y = 0,1084x + 6,6074	0,98661
	RNA 2-	y = 0,6426x + 5,20	0,8981#	y = 0,1032x + 5,7019	0,91588#
r1412	RNA 1	y = 0,7919x + 3,8249	0,90157#	y = 0,1412x + 3,9679	0,99608
	RNA 2+	y = 0,8099x + 6,1833	0,94239#	y = 0,1183x + 6,9295	0,92830#
	RNA 2-	y = 0,6901x + 5,6052	0,87872#	y = 0,1204x + 5,8837	0,89729#
r1408-1412	RNA 1	y = 0,5714x + 4,2606	0,90837#	y = 0,0821x + 4,5872	0,93404#
	RNA 2+	y = 0,7933x + 5,911	0,96755	y = 0,1167x + 6,597	0,98989
	RNA 2-	y = 0,7169x + 5,3071	0,94907#	y = 0,1122x + 5,7299	0,97779

The extension of the exponential phase of the RNA synthesis was searched analysing its kinetics of production by regression analysis. The extension best fitting a line was considered to constitute the exponential phase. R^2 values with pound signs correspond to data not adjusting to a reliable regression line.

Table S2.- Regression analysis: Kinetics curves of RNA synthesis-Correlation between RNA1 and RNA 2, and $RNA2^+$ and $RNA2^-$

		Regression	R^2	P (RNA1/2+)	P (RNA2-/2+)
Wt160	RNA 1	$y = -0{,}0011x^2 + 0{,}1086x + 5{,}4012$	0,97148	0.1020	
	RNA 2+	$y = -0{,}0017x^2 + 0{,}1308x + 7{,}1823$	0,98668		0.0475*
	RNA 2-	$y = -0{,}001x^2 + 0{,}0945x + 6{,}3611$	0,95455		
r160	RNA 1	$y = -0{,}0008x^2 + 0{,}0956x + 5{,}2713$	0,96528	0.0514	
	RNA 2+	$y = -0{,}0015x^2 + 0{,}1288x + 6{,}7283$	0,99955		0.2207
	RNA 2-	y = -0,0018x2 + 0,1407x + 5,8115	0,95082		
r1408	RNA 1	$y = -0{,}0005x^2 + 0{,}0836x + 4{,}6313$	0,95908	0.0175*	
	RNA 2+	$y = -0{,}0017x^2 + 0{,}1414x + 6{,}6311$	0,98486		0.1173
	RNA 2-	$y = -0{,}002x^2 + 0{,}1466x + 5{,}6559$	0,96817		
r1412	RNA 1	$y = -0{,}0013x^2 + 0{,}1277x + 4{,}6351$	0,90312#	0.4310	
	RNA 2+	y = -0,0021x2 + 0,167x + 6,8369	0,97969		0.2829
	RNA 2-	$y = -0{,}0017x^2 + 0{,}1401x + 6{,}1693$	0,91627#		
r1408-1412	RNA 1	$y = -0{,}0004x^2 + 0{,}0663x + 4{,}9692$	0,93126#	<0.0001***	
	RNA 2+	$y = -0{,}0019x^2 + 0{,}1542x + 6{,}5983$	0,98690		0.1735
	RNA 2-	y = -0,0013x2 + 0,124x + 5,9984	0,95907		

With pound signs: R^2 values below 0.95

Table S3.- Comparison between Kinetics curves of RNA synthesis

	RNA 1 curves comparison				
	wt160	r160	r1408	r1412	r1408-1412
wt160		H_0 P=6735	H_0 P=2579	H_0 P=5506	H_0 P=1246
r160	H_a*** P<0.0001		H_0 P=6230	H_0 P=4688	H_0 P=2780
r1408	H_a* P=0.0074	H_0 P=4997		H_0 P=3459	H_0 P=4057
r1412	H_a*** P=0.0003	H_a* P=0.0111	H_0 P=1772		H_0 P=0925
1408-1412	H_a*** P<0.0001	H_a* P=0.0124	H_0 P=3126	H_0 P=7285	
	RNA 2 curves comparison				

H_0: null hypothesis is not rejected. H_a: alternative hypothesis (null hypothesis is rejected).

Fig. S1 Regression analysis of the curves of viral production

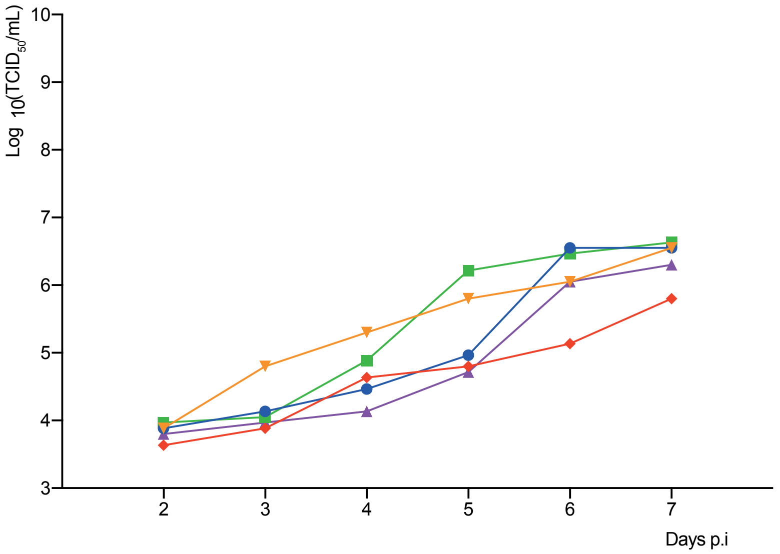

Regression analysis[1]

- wt160 $y = 0.6024x + 2.3810$ $R^2 = 0.8408$
  $y = 0.0430x_2 + 0.3919x + 0.8040$ $R^2 = 0.8630$
- r160 $y = 0.4214x + 2.7510$ $R^2 = 0.8761$
  $y = 0.0280x_2 + 0.2548x + 0.5227$ $R^2 = 0.8767$
- r1408 $y = 0.6262x + 2.5520$ $R^2 = 0.9009$
  $y = 0.0356x_2 + 0.3249x + 0.6667$ $R^2 = 0.9066$
- r1412 $y = 0.5524x + 2.3420$ $R^2 = 0.8906$
  $y = 0.0250x_2 + 0.2273x + 0.4663$ $R^2 = 0.9419$
- r1408-1412 $y = 0.5024x + 3.137$ $R^2 = 0.9624$
  $y = 0.0103x_2 + 0.0937x + 0.1923$ $R^2 = 0.9871$

Fig. S2 Regression analysis of the RNA1 production curves

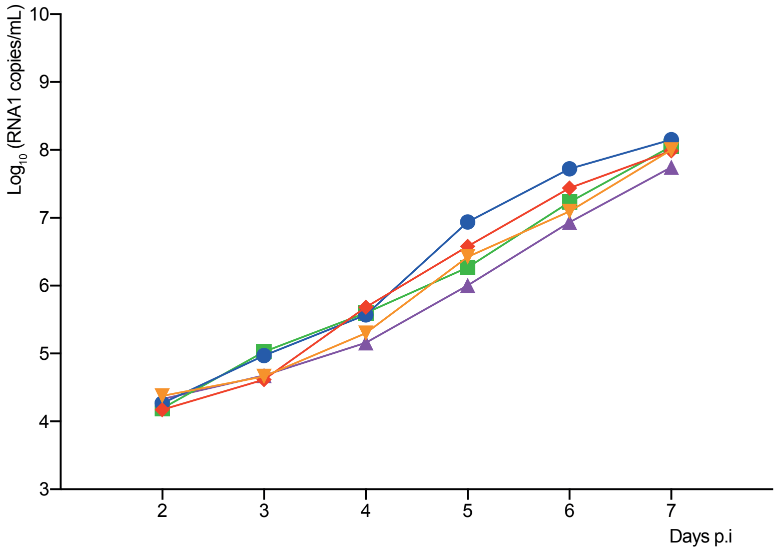

Regression analysis[2]

- wt160 $y = 0.8118x + 2.427$ $R^2 = 0.9897$
- r160 $y = 0.8299x + 2.536$ $R^2 = 0.9808$
- 1408 $y = 0.7593x + 2.646$ $R^2 = 0.9942$
- 1412 $y = 0.7053x + 2.634$ $R^2 = 0.9737$
- 1408-12 $y = 0.7582x + 2.565$ $R^2 = 0.9777$

P[3]

	wt160	r160	r1408	r1412	r1408-1412
wt160	-	0.8062	0.3293	0.1727	0.4690
r160		-	0.3084	0.1670	0.4844
r1408			-	0.4287	0.9864
r1412				-	0.5341
r1408-1412					-

Fig. S3 Regression analysis of the RNA2 production curves

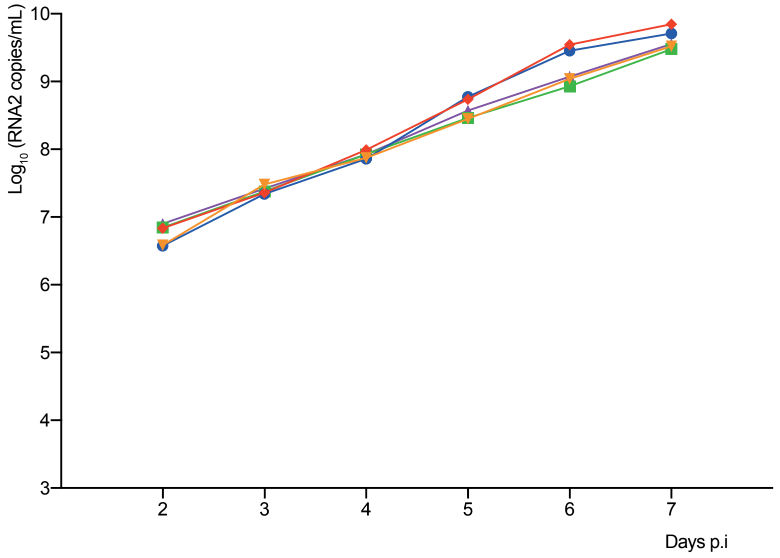

Regression analysis

- wt160 $y = 0.6388x + 5.510$ $R^2 = 0.9901$
- r160 $y = 0.6546x + 5.339$ $R^2 = 0.9839$
- r1408 $y = 0.5246x + 5.809$ $R^2 = 0.9995$
- r1412 $y = 0.5389x + 5.816$ $R^2 = 0.9985$
- r1408-1412 $y = 0.5689x + 5.589$ $R^2 = 0.9898$

P

	wt160	r160	r1408	r1412	r1408-1412
wt160	-	0.7722	0.0079**	0.0180*	0.1431
r160		-	0.0152*	0.0280*	0.1302
r1408			-	0.2688	0.1702
r1412				-	0.3574
r1408-1412					-

1- Second degree polynomial regression.
2- Lineal regression
3- Comparison of curves.-Pvalues considered significant: <0.05 (*, significant), 0.05-0.01 (**, very significant), <0.0001 (***, extremely significant)
